# Genes with Restricted Introgression in a Field Cricket (*Gryllus firmus/Gryllus pennsylvanicus*) Hybrid Zone Are Concentrated on the X Chromosome and a Single Autosome

**DOI:** 10.1534/g3.115.021246

**Published:** 2015-08-26

**Authors:** Luana S. Maroja, Erica L. Larson, Steven M. Bogdanowicz, Richard G. Harrison

**Affiliations:** * Department of Biology, Williams College, Williamstown, Massachusetts 01267; † Division of Biological Sciences, University of Montana, Missoula, Montana 59812; ‡ Department of Ecology and Evolutionary Biology, Cornell University, Ithaca, New York 14853

**Keywords:** genetic map, mapping family, backcross, sex chromosome, speciation

## Abstract

Characterizing the extent of genomic differentiation between recently diverged lineages provides an important context for understanding the early stages of speciation. When such lineages form discrete hybrid zones, patterns of differential introgression allow direct estimates of which genome regions are likely involved in speciation and local adaptation. Here we use a backcross experimental design to construct a genetic linkage map for the field crickets *Gryllus firmus* and *Gryllus pennsylvanicus*, which interact in a well-characterized hybrid zone in eastern North America. We demonstrate that loci with major allele frequency differences between allopatric populations are not randomly distributed across the genome. Instead, most are either X-linked or map to a few small autosomal regions. Furthermore, the subset of those highly differentiated markers that exhibit restricted introgression across the cricket hybrid zone are also concentrated on the X chromosome (39 of 50 loci) and in a single 7-cM region of one autosome. Although the accumulation on the sex chromosome of genes responsible for postzygotic barriers is a well-known phenomenon, less attention has been given to the genomic distribution of genes responsible for prezygotic barriers. We discuss the implications of our results for speciation, both in the context of the role of sex chromosomes and also with respect to the likely causes of heterogeneous genomic divergence. Although we do not yet have direct evidence for the accumulation of ecological, behavioral, or fertilization prezygotic barrier genes on the X chromosome, faster-X evolution could make these barriers more likely to be X-linked.

Characterizing the genomic architecture of barriers to gene exchange is an essential step in understanding speciation. It is well established that because of independent assortment and recombination in sexually reproducing organisms, genome regions will have unique evolutionary histories (Hudson 1992; Nichols 2001). Furthermore, if barriers to gene exchange are a result of evolutionary forces acting on individual genes (genic view of speciation) (Wu 2001), then in species that still exchange genes, alleles that are neutral or globally advantageous will be able to cross species boundaries, whereas alleles in genome regions under divergent selection or responsible for reproductive isolation will not. The result is that species boundaries are semipermeable (Barton and Hewitt 1981; Harrison 1990; Wu 2001). In some instances only a few loci may be essential for the maintenance of species differences (*e.g.*, Machado and Hey 2003; Dopman *et al.* 2005; Barr and Fishman 2010; Nosil and Schluter 2011; Dasmahapatra *et al.* 2012), and it has been suggested that, in the face of gene flow, these loci might act as “divergence centers” (Charlesworth *et al.* 1997), leading to the buildup of additional divergence because of reduced local recombination rates (divergence hitchhiking). With time this process is expected to lead to a clustering of loci contributing to differentiation (Charlesworth *et al.* 1997; Strasburg *et al.* 2012)

Genome scans have identified divergent genomic regions in many recently diverged, hybridizing species (*e.g.*, Nosil *et al.* 2009; Carneiro *et al.* 2010; Nadeau *et al.* 2012), a pattern that has been interpreted as islands of restricted introgression in a background of relatively free gene exchange [“genomic islands of speciation” (Turner *et al.* 2005) or “genomic islands of differentiation” (Harr 2006; Nosil *et al.* 2009)]. Recently, however, this interpretation has been challenged (Nachman and Payseur 2012; Cruickshank and Hahn 2014) because the same pattern of heterogeneous genomic divergence could occur in the complete absence of gene flow. In this scenario, many genome regions would be characterized by shared ancestral polymorphism, but other (differentiated) regions would have been subject to local selective sweeps.

By definition, hybrid zones are regions where hybridization and introgression can occur. Therefore, hybrid zones are natural experiments in which genomes are recombined and subjected to selection over many generations (Hewitt 1988; Harrison 1990; Payseur 2010). Thus, for each gene, the nature and strength of selection, the role in reproductive isolation and local adaptation, and linkage to other genes will determine the extent of introgression. Although variation in the extent of introgression among loci has been well documented (reviewed in Harrison and Larson 2014), patterns of introgression have been placed in the context of a genetic or physical map in relatively few cases (Rieseberg *et al.* 1999; Payseur *et al.* 2004; Noor *et al.* 2007; Janousek *et al.* 2012; Sankararaman *et al.* 2014).

Here we construct a genetic linkage map to provide the framework for evaluating patterns of differential introgression across the hybrid zone between the field crickets *Gryllus firmus* and *Gryllus pennsylvanicus*. This species pair is recently diverged (∼200,000 years) (Broughton and Harrison 2003; Maroja *et al.* 2009a), and multiple barriers to gene exchange have been well characterized. These barriers include: (1) habitat isolation (*G. firmus* lives in sandy soils and *G. pennsylvanicus* lives in loamy soils) (Rand and Harrison 1989; Ross and Harrison 2002; Ross and Harrison 2006; Larson *et al.* 2013b); (2) temporal isolation (*G. firmus* matures later in the summer in parts of the hybrid zone) (Harrison 1985); (3) behavioral and cuticular hydrocarbon differences that result in assortative mating (Maroja *et al.* 2009b, 2014); and (4) a postmating prezygotic barrier in which *G. pennsylvanicus* sperm are unable to fertilize *G. firmus* eggs (Harrison 1983; Larson *et al.* 2012b). The last barrier results in a unidirectional reproductive incompatibility (only *G. pennsylvanicus* females can produce F1 hybrids). Although these barriers substantially reduce gene flow between the two species, hybridization, and introgression are ongoing. F1 hybrids are rare, but many sites within the hybrid zone include crickets that represent multi-generation backcrosses; using crickets from allopatric populations as reference, we have argued that shared ancestral polymorphism cannot explain observed patterns (Harrison and Bogdanowicz 1997; Larson *et al.* 2013a, 2014).

Two locations within the hybrid zone (Connecticut and Pennsylvania) have been characterized for differential introgression (Larson *et al.* 2013a, 2014). A total of 110 SNP markers were analyzed at both locations and an additional 15 were analyzed only in the Pennsylvania hybrid zone. These markers were developed from comparison of male accessory gland transcriptomes (Andres *et al.* 2013) and were chosen because they represent SNPs that exhibit nearly fixed differences between allopatric populations of the two cricket species. Patterns of introgression were consistent between locations; 50 of the 110 markers exhibited reduced introgression. The repeatability of this pattern suggests that selection is acting to reduce introgression, and thus these regions are likely to include genes contributing to reproductive isolation or local adaptation. Here we demonstrate that the loci with major allele frequency differences between allopatric populations are not randomly distributed across the genome. Instead, most are either X-linked or map to a few small autosomal regions. Furthermore, the subset of those differentiated markers that exhibit reduced introgression are also concentrated on the X chromosome (39 of the 50 loci) and in a single 7-cM region of one autosome (LG14). We discuss the implications of these results for speciation and divergence in hybridizing species.

## Materials and Methods

### Mapping panel

**Figure 1:**
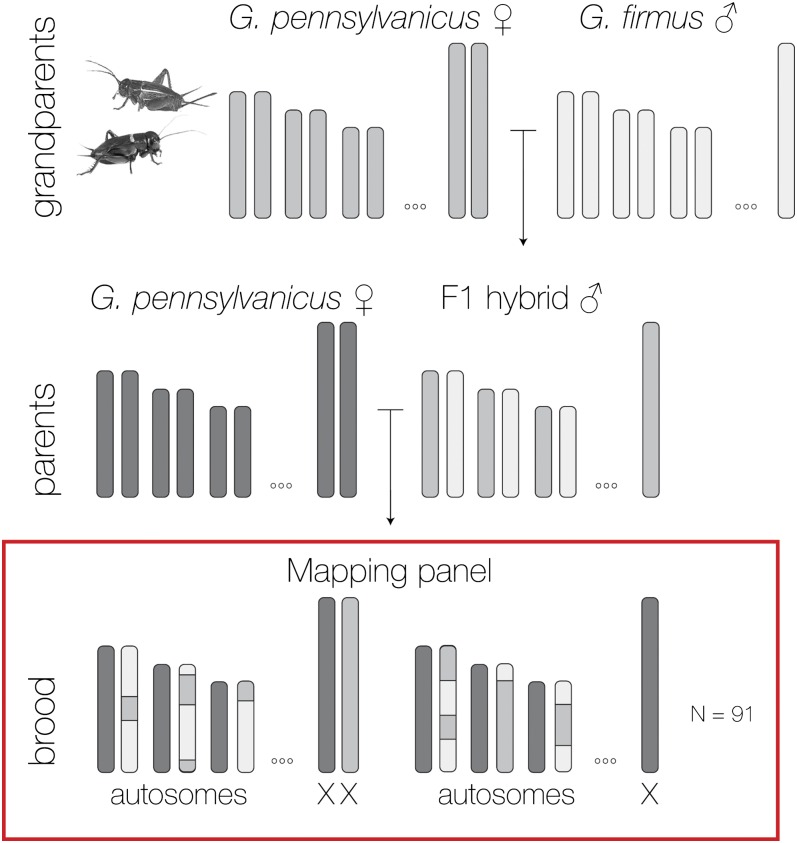
Diagram of the cricket mapping family highlighting segregation of autosomes and the X chromosome. Chromosomes from *G. firmus* are shown in white and chromosomes from *G. pennsylvanicus* are shown in two gray tones to highlight that the lines were not inbred.

Because *Gryllus pennsylvanicus* sperm fail to fertilize *Gryllus firmus* eggs, we produced F1 hybrids from a cross of *G. pennsylvanicus* female × *G. firmus* male and then backcrossed an F1 male to a *G. pennsylvanicus* female to obtain a mapping panel of 91 offspring (Figure 1). Parental individuals were collected from Guilford, Connecticut (41°15′, −72°42′) and from Ithaca, New York (42°24′, −76°31′) in the fall of 2011. All individuals were raised with *ad libitum* food (cat and rabbit food) and water at a controlled temperature of 30°, 70% humidity, and 12:12 light/dark cycle. Petri dishes with a combination of potting soil and sand were provided as oviposition substrate, and eggs were incubated at 4° for 3 months to break diapause and guarantee synchronous hatch. Offspring were collected within 2 d of hatching. DNA from parents and offspring was extracted with Qiagen DNeasy tissue kits. In total we analyzed 91 offspring, the two parents, and two additional F1 females (full sisters of the F1 male).

### Multiplex PCR of highly differentiated RNAseq loci

To genotype the single nucleotide polymorphisms (SNPs) from highly differentiated RNAseq loci that were previously characterized across the hybrid zones (Larson *et al.* 2013a, 2014) we set up multiplex PCR reactions, each containing 21–25 primer pairs (primers described in Larson *et al.* 2013a). We used the Qiagen Multiplex PCR Kits following manufacturer recommendations, with the following adjustments (recommended for more than 10 PCR products): our primer concentration was decreased to a final concentration of 0.1 μM and the annealing time was increased to 3 min in a total volume of 10 μl. We set up five multiplex PCRs for each individual and then combined the resulting five PCR products. We cleaned up the combined multiplex PCR reactions by precipitation, adding 3 μl of combined PCR reaction to 3 μl of sodium acetate (3 M) and 55 μl of 100% ethanol. We placed the solution at −20° for 20 min and then centrifuged the solution at maximum speed for 10 min. The resulting pellet was resuspended in 14 μl of EB buffer (Qiagen). To ensure ligation of Illumina TruSeq adaptors, we adenylated the cleaned PCR products with Klenow fragment (exo^−^) (New England Biolabs), adding 1 μl of NEB #2 buffer, 1 μl of dATP (1 mM), 0.5 μl of enzyme, and 2 μl of PCR product in a total volume of 10 μl. We then ligated adaptors, adding 4 μl of NEB #2 buffer, 1 μl of dsAdaptor (+A), 1.4 μl of ATP (10 mM), and 1 μl of T4 DNA ligase (New England Biolabs) to each tube and incubating at room temperature for 1 hr. Finally, we added the individual specific Illumina index barcodes by PCR (platinum taq DNA polymerase; Life Technologies) using the Illumina Index primer and the universal forward primer in a total volume of 25 μl (27 cycles with 60° annealing temperature). After addition of barcodes, DNAs from all individuals were combined (1 μl per individual) and the solution was cleaned with Agencourt AMPure XL beads (Beckman Coulter, Inc.) at 1.8× concentration. We ran 48 individuals per lane (two lanes) on an Illumina Hisequation 2500 with paired end sequencing (2×150 bp) (Cornell Life Sciences Core Laboratory Center). We analyzed reads in Geneious R7 (Biomatters) using the known sequences as a “reference” to which reads were mapped.

### ddRAD

We used ddRAD sequencing (Peterson *et al.* 2012) to identify additional SNPs, which could be used as markers in constructing the genetic map. We used *Msp*I (New England Biolabs) as the common cutter and SbfI (New England Biolabs) as the rare cutter and followed library construction steps as in Peterson *et al.* (2012). We used two Illumina HiSeq lanes to sequence 93 barcoded individuals at the Cornell Life Sciences Core Laboratory Center. We used Geneious R7 (Biomatters) to separate barcodes and trim adaptors and Ngen v11 (DNAstar) to build a *de novo* reference (based on two parents) and to assemble all offspring to the reference. We then used Arraystar v11 (DNAstar) to call SNPs that were genotyped successfully in a minimum of 80 individuals with a minimum read depth of 10 and a minor allele frequency of at least 0.2. After checking for Mendelian segregation and eliminating loci that deviated strongly, we visually inspected every parental alignment and further eliminated all loci that had more than two haplotypes per individual/contig.

To increase the density of markers on the X chromosome, we regenotyped 51 SNPs from the ddRAD data that had low individual coverage (and were thus excluded from the ddRAD data), no evidence of paralogy, and were segregating in the *G. pennsylvanicus* mother. We developed primers that flanked each SNP using BatchPrimer3 (Untergasser *et al.* 2012), and to the 5′ end of each forward and reverse primer we added Illumina Nextera adaptor sequences (TCGTCGGCAGCGTCAGATGTGTATAAGAGACAG and GTCTCGTGGGCTCGGAGATGTGTATAAGAGACAG, respectively). A total of 45 loci had strong PCR products and were amplified by multiplex PCR (Qiagen Multiplex PCR Kit) in three separate mixes. We pooled the three resulting PCR product pools for each individual and added Illumina dual i5/i7 barcodes by PCR (OneTaq; New England Labs). Finally, we pooled all individuals in a single mix and cleaned the reaction with 1.6× Agencourt AMPure XL beads (Beckman Coulter, Inc.). The resulting data were analyzed in the same way as the other multiplex PCR data (see *Multiplex PCR of highly differentiated RNAseq loci*).

### Microsatellites

We also genotyped the mapping family for 15 microsatellites, 10 that had previously been characterized in Larson *et al.* (2012a) and Larson *et al.* (2012b) (accession numbers JN375323– JN375329; JN375360; JX050156–JX050157) and five that were unpublished (accession numbers: KM203912–KM203916; see Supporting Information, Table S2 for further information). We used Qiagen Type-it microsatellite kit following manufacturer recommendations to run three multiplex PCRs. Each reaction contained five loci labeled with four different fluorescent dyes (Fam, Vic, Pet, and Ned). Two loci in each reaction mix were labeled with the same dye but had nonoverlapping product size. We diluted PCR products 1:15 in water and then mixed 1.5 μl of dilution with 18 μl of Hi-Di Formamide (Applied Biosystems, Foster City, California, USA) and 0.15 μl of GeneScan 500 LIZ Size Standard (Applied Biosystems). Samples were genotyped at the Cornell Life Sciences Core Laboratory Center on an Applied Biosystems 3730xl DNA Analyzer. We analyzed electropherograms with GeneMarker v2.4.0 (Softgenetics).

### Map construction

Segregating loci that did not deviate strongly from Mendelian expectation were kept in the analyses. Many of the X-linked loci could not be ordered because our cross (F1 male backcrossed to *G. pennsylvanicus* female) had few informative sites on the X chromosome (male crickets are XO, and thus F1 males have no recombination on the X chromosome). However, for the highly differentiated RNAseq loci that were known to be fixed between species (Andres *et al.* 2013; Larson *et al.* 2013a, 2014), we were able to infer X-linkage from a pattern in which F1 females (sister to the F1 father) are heterozygous (expected if parents are fixed for different alleles) and the F1 male (father) is homozygous (confirming X-linkage). Our mapping family was classified as the CP population type (outbreeding population full-sibling family, raw JoinMap data in Table S1) implemented in JoinMap 4.0 (Van Ooijen 2006), where maternal segregation is coded as “lm × ll,” paternal segregation is coded as “nn × np,” and segregation in both parents is coded as “hk × hk,” “ef × eg,” or “ab × cd” (the last two segregation patterns were only observed in microsatellites). To evaluate any discrepancy from the expected segregation ratios we used the χ^2^ goodness-of-fit method as implemented in JoinMap 4.0. All ddRAD markers showing segregation distortion at the significance level of *P* = 0.01 were eliminated from the analyses (highly differentiated RNAseq loci and microsatellites did not show deviations). Linkage groups were determined using a logarithm of odds (LOD) threshold of 4.0. We used the Kosambi mapping function and the regression mapping algorithm for map construction (Haldane function yielded similar results, not shown).

The highly differentiated RNAseq loci, developed from a transcriptome library, represent markers that had large allelic differences between species. To test if these markers were randomly distributed across the genome, we fit the number of markers per chromosome to a Poisson distribution (Zar 1999). We also tested whether these markers were randomly distributed within chromosomes by dividing each chromosome into 10-cM bins and fitting the data to a Poisson distribution (Zar 1999). Using R (R Core Team 2013), we simulated possible marker positions along the chromosome assuming a uniform distribution and then compared observed and simulated average distances between markers.

### Data availability

The genotype dataset used to build the genetic map is available as Supplementary Information (Table S1). Information about new microsatellite markers developed for this project (including repeat motif, primers and accession numbers) can be found in Table S2. Locus specific information, including interspecific differentiation index, introgression across both hybrid zones, and position on the map, can be found in Table S3. Raw Illumina reads from ddRAD and multiplex PCR are available upon request.

## Results

**Figure 2:**
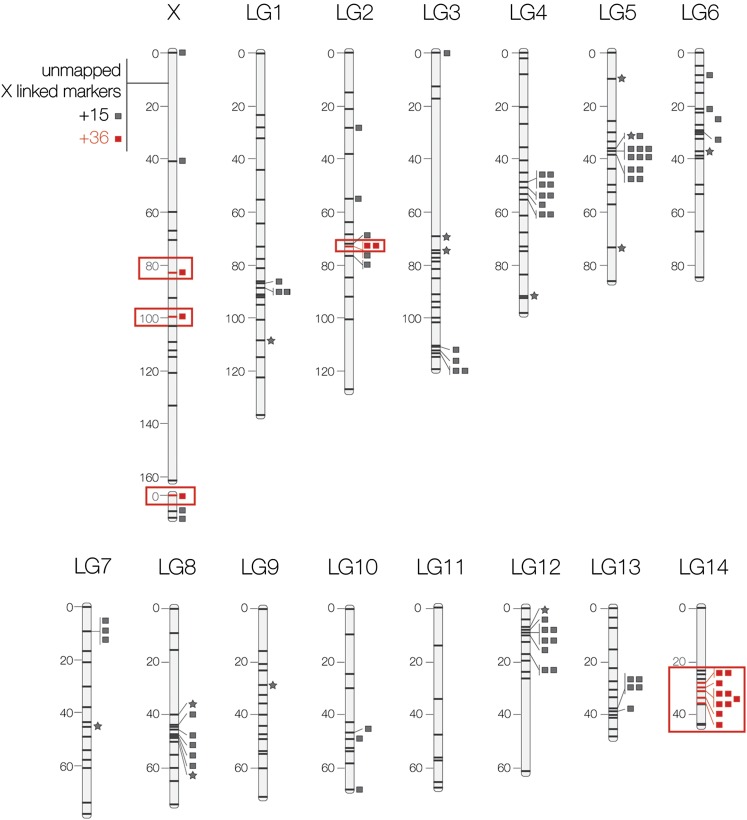
Linkage map including ddRAD markers (unmarked bars), previously characterized loci (squares), and microsatellite markers (stars). Markers highlighted in red showed reduced introgression across the hybrid zone (Larson *et al.* 2014).

We recovered the 15 linkage groups (LG) known from the *Gryllus pennsylvanicus* karyotype (Lim *et al.* 1973) (see Figure 2). In addition, three X-linked markers formed their own LG (*i.e.*, were not joined together with other X-linked markers). The total map length was 1335.7 cM, based on 276 markers across the genome (179 ddRADs, 13 microsatellites, 84 highly differentiated RNAseq loci); an additional 51 loci were placed on the X chromosome but not ordered on the map. Below we provide more detailed results for the three types of markers that we used for our map. These include markers identified from RNAseq that show major allele frequency differences between allopatric populations of the two cricket species, as well as the subset of those markers that exhibit restricted introgression across the hybrid zone.

### Microsatellites

Of the 15 microsatellites, 13 were mapped. One of the excluded microsatellites had a very weak signal and could not be reliably genotyped (Gr145), and the other had the same combination of a recognizable allele and a null allele in each of the parents, making it impossible to tell a homozygote (non-null) from a heterozygote (with null) individual (Gr54). Except for one locus (G3, homozygous in female parent), all other microsatellites were segregating in both parents and had high levels of amplification success (0.3% missing data) and quality (no ambiguous calls). The 13 mapped microsatellites were randomly distributed both within and across linkage groups (χ^2^_1_= 0.27, *P* > 0.5).

### ddRADs

We obtained 837 SNPs from the ddRAD assembly, but 437 (52.2%) were immediately eliminated because they did not meet the expectations of Mendelian segregation (*P* < 0.0001). We visually inspected individual read alignments from the F1 father for the remaining 400 loci and eliminated a further 81 SNPs (18.3%) from contigs that we assumed included paralogs, that is, contigs with three or more haplotypes per individual (no single SNP position had more than two alleles, but there were multiple haplotypes within the contig of a single individual). The remaining 319 ddRAD loci had 4.5% missing data.

Of the 319 SNPs, we mapped 157. We eliminated 162 loci because they had identical map positions to other loci (n = 151) or did not map to any LG (n = 11). Because only eight ddRAD loci mapped to the X chromosome, we examined additional ddRAD loci that were female segregating but did not have enough coverage to be included in our initial sample (see *Materials and Methods*). We then genotyped 45 ddRAD SNPs; of these, 11 appeared to include paralogs, four were monomorphic, had map positions identical to other loci (n = 6), or did not map to any LG (n = 2). Of the additional 22 loci that we were able to map, three were X-linked.

In sum, we mapped 179 SNPs identified from ddRAD sequences. These SNPs were randomly distributed both within and across linkage groups (χ^2^_18_= 8.94; *P* > 0.5).

### Highly differentiated RNAseq loci

We genotyped the parents (F1 male and *G. pennsylvanicus* female), 91 backcross offspring, and two F1 females (sisters of the F1 male) for 141 loci developed from the RNAseq library (136 unique highly differentiated RNAseq loci and an additional five loci not characterized across the hybrid zone) (Table S3). Of these we were able to place 84 loci on the map, seven of them on the X chromosome. In addition, we could assign an additional 51 loci to the X chromosome (see below), but because of lack of recombination in the F1 male, these markers could not be ordered along the chromosome. The remaining six loci were not mapped either because the whole family was heterozygous (two loci; mother and father homozygous for alternative alleles) or because the locus was completely monomorphic across all genotyped individuals (four loci).

After separating individuals by barcode and trimming regions with an error probability higher than 5%, the multiplex PCR approach yielded high coverage data with a mean (± SD) of 466,547 ± 240,100 paired end reads per individual and mean read length of 84.7 ± 10 bp per read. We aligned these data to a reference library of the known sequences from the transcriptome based RNAseq loci (Andres *et al.* 2013; Larson *et al.* 2013a, 2014). The aligned contigs had an average mean coverage of 1578.6 ± 1427.8 per contig position. To call SNPs we produced consensus sequences for each locus/individual. We recorded ambiguities (*i.e.*, S, W, Y, R, K, M) at positions with minor allele frequency of at least 25% and a minimum coverage of eight reads (individuals with low coverage were visually inspected and assigned as “missing” if data were not high-quality). We then realigned the consensus sequences to the reference and called SNPs. Most SNPs matched the expected positions (previously characterized), although in a few cases additional SNPs were also segregating. Discounting four loci with too much missing data (complete failure in first or second Illumina lane), there was a total of 3% missing data (with two individuals contributing to most of the missing data).

Although the backcross offspring could not be sexed directly, we were able to assign sex using segregation patterns at loci where the F1 father and the *G. pennsylvanicus* mother were homozygous for alternative alleles. An autosomal inheritance model would yield only heterozygous offspring, but for some loci we observed that the brood segregated in a nearly 1:1 ratio (heterozygote:homozygote for maternal allele), suggesting that these loci were sex-linked. Assuming X-linkage, all females should be heterozygotes and all males should inherit only the maternal allele. We confirmed sex assignment with multiple X-linked loci. Using these observations, we identified 40 females and 51 males in the mapping family.

Most X-linked loci were not segregating in the mapping family, because the F1 parent was a hemizygous father carrying a *G. pennsylvanicus* X chromosome. Nonetheless, we were still able to identify which highly differentiated RNAseq loci were X-linked. These loci were nearly fixed for different alleles between species and thus unlikely to be homozygous in an F1 female. Therefore, a pattern in which the F1 male parent of the mapping family appears homozygous, his F1 full sisters are heterozygous, and the *G. pennsylvanicus* allele is fixed in the backcross family is most parsimoniously explained by X-linkage. The only alternative explanation for this pattern would be if autosomal *G. pennsylvanicus* alleles were segregating in the population of the *G. firmus* grandfather, leading to homozygosity for the *G. pennsylvanicus* allele in the F1 male (father) and heterozygosity in the two F1 females (his sisters). This alternative scenario has probabilities ranging from 0% (in the 45% of the postulated X-linked markers for which alternative alleles are fixed between species) to <2.5% for another 45% of the markers (when the two species are nearly fixed for alternative alleles) to a maximum of <6% for a single locus (where *G. pennsylvanicus* is fixed for an allele and that allele is segregating in *G. firmus* populations at a frequency of ∼0.3). Based on these arguments, we assigned a total of 58 loci to the X chromosome, but 51 could not be ordered on the map (Figure 2).

The distribution of the highly differentiated RNAseq loci across the genome was not random. A disproportionate fraction of the mapped markers was located on the X chromosome (43.9% of markers, χ^2^_56_= 1.7×10^26^, *P* < 0.0001). Excluding the X chromosome, the distribution across autosomes was also clumped (χ^2^_12_= 68.61; *P* < 0.0001), with more than expected chromosomes with zero markers (n = 2) and one chromosome with 12 markers. Finally, markers were not randomly distributed across the length of each chromosome; for most autosomes, loci were highly clustered (*P* < 0.01; *P* value obtained from nearest neighbor data simulation), with the exception of LG6 (*P* = 0.21) and LG10 (*P* = 0.34). In contrast, the seven mapped markers on the X chromosome were randomly distributed (*P* = 1).

We mapped 114 of the 120 unique highly differentiated loci analyzed for introgression across the Pennsylvania (Larson *et al.* 2013a) hybrid zone and 105 of the 110 loci analyzed across the Connecticut hybrid zone (Larson *et al.* 2014) (markers analyzed across the hybrid zones are the same, but a reduced subset was analyzed in Connecticut). The two hybrid zones were concordant with respect to patterns of introgression—only one marker showed inconsistent genomic cline patterns between the two regions, but the Pennsylvania hybrid zone had fewer individuals and thus less power, resulting in fewer markers showing significantly reduced introgression. In the Pennsylvania hybrid zone, all loci with significantly reduced introgression (n = 33) mapped to the X chromosome (25 of out of 55 total X-linked loci), to LG14 (nine out of nine LG14 linked loci), or to LG5 (one out of 10 LG5 linked loci; this single locus with reduced introgression was only analyzed in the Pennsylvania hybrid zone). For the subset of loci analyzed in the Connecticut hybrid zone, all loci with reduced introgression (n = 50) mapped to the X chromosome (39 of 49 X-linked loci), to LG14 (nine out of nine LG14 linked loci), or to LG2 (two out of five LG2 linked loci; the two reduced introgression loci did not show significantly reduced introgression in the Pennsylvania hybrid zone).

## Discussion

Our map data reveal a distinctly nonrandom distribution of markers that exhibit major allele frequency differences between cricket species and/or show significantly reduced introgression across the hybrid zone. The 50 loci that have reduced introgression between the recently diverged hybridizing field crickets *Gryllus firmus* and *G. pennsylvanicus* (Larson *et al.* 2014) map either to the sex chromosome or to LG14. Furthermore, previously characterized highly differentiated RNAseq loci (Larson *et al.* 2013a, 2014) cluster within chromosomes and are particularly concentrated on the X chromosome. Below we discuss implications of these results for understanding the speciation process.

### Highly differentiated loci are clustered in the field cricket genome

We recovered the 15 LGs (chromosomes) expected in *Gryllus* (Lim *et al.* 1973) and were able to order and map (n = 79) or localize to the X chromosome (n = 51) most of the previously characterized highly differentiated RNAseq loci (see Figure 2). All of these previously characterized loci represent coding regions with fixed or nearly fixed differences between species (allele frequency difference of at least *D* > 0.8) (Andres *et al.* 2013). These genes clustered both among chromosomes (43.9% X-linked, *P* < 0.0001) (Figure 2) and within chromosomes (genes are in close linkage within most LGs, *P* < 0.01, except in LG6 and LG10).

The RNAseq loci we mapped represent protein coding genes expressed in the male accessory gland (Andres *et al.* 2013; Larson *et al.* 2013a). Although coding regions could be nonrandomly distributed across the cricket genome, the extent of clustering both among and within chromosomes suggests that other explanations are more likely. For example, clustering might be better explained if regions of high differentiation experienced recent positive selection in one or both species, leading to decreased diversity and high differentiation (or fixed differences) between species. Genetic hitchhiking would lead to decreased genetic diversity in nearby genes causing the entire region to become highly differentiated. Genes within the high differentiation cluster would then be chosen for study because of their high differentiation or fixed differences (Larson *et al.* 2013a, 2014).

Although the X is the largest cricket chromosome (Lim *et al.* 1973), representing approximately 20% of the genome, even after correcting for size the X is still enriched with highly differentiated loci. It is clear that selection acts differently on the X compared to autosomes. X-linked loci may evolve faster than autosomal loci simply due to differences in effective population size (3/4 N_e_). Smaller N_e_ may lead to more rapid coalescence and greater exclusivity, thus making it more likely that the highly differentiated loci would be X-linked. If new advantageous loci are on average recessive, then selection may be more effective on the X because recessive alleles are exposed to selection in hemizygous males (Charlesworth *et al.* 1987; reviewed in Meisel and Connallon 2013). Thus, faster-X evolution could also lead to the more rapid accumulation of highly differentiated loci between species. Finally, because of its hemizygosity, the X recombines only in females, resulting in tighter linkage compared to autosomes. Thus, even if only a few genes are under selection, many other genes could hitchhike with them. This process would reduce diversity, making the accumulation of X-linked highly differentiated loci more likely (reviewed in Qvarnstrom and Bailey 2009).

The SNPs we identified from RNAseq data reside in gene regions that were originally isolated from comparison of accessory gland transcriptome libraries (Andres *et al.* 2013). These libraries were generated using pooled DNA from 15 males of each species. Because males have only one X chromosome, effective sample sizes for X-linked loci would be half that of autosomal loci, potentially leading to an excess of highly differentiated X linked loci in our first screen. The accessory gland in crickets is a tissue unique to males. Genes with sex biased expression are often either overrepresented or underrepresented on the X in a taxon-specific manner (Meisel *et al.* 2012). In *Drosophila*, genes expressed in reproductive tissues are under-represented on the X (Wolfner *et al.* 1997; Swanson *et al.* 2001; Mueller *et al.* 2005), whereas in mammals such genes are overrepresented on the X (Lercher *et al.* 2003; Khil *et al.* 2004; Meisel *et al.* 2012). If crickets have an overrepresentation of male-specific genes on the X, this might also contribute to the observed high proportion of X-linked highly differentiated RNAseq loci.

### Restricted introgression of loci on the X and one autosome

The X chromosome was enriched for highly differentiated markers, and the majority of genes with reduced introgression across the hybrid zone (39 out of 50) (Larson *et al.* 2014) were also X-linked (Figure 2). Although most X-linked loci could not be ordered along the X (51 out of 58), the mapped markers on the X did not have a clumped distribution like that observed for autosomes. Even the three ordered genes with reduced introgression were not in close linkage on the X (Figure 2), suggesting that the entire X chromosome, and not simply a few regions of the X, may be enriched for both high-differentiation loci and for loci with reduced introgression across the hybrid zone.

The large role of the X in speciation has been well documented for genes contributing to hybrid sterility and inviability (reviewed in Presgraves 2008). In addition to faster-X evolution, the other potential mechanisms behind X-linked sterility are mis-regulation of the X chromosome during spermatogenesis (Lifschyt and Lindsley 1972; Masly and Presgraves 2007), gene movement on and off the X (Moyle *et al.* 2010), or dominance (recessive deleterious alleles are exposed in the male’s single X) (Turelli and Orr 1995). However, we have no evidence of abnormal spermatogenesis in F1 hybrid male crickets; although they cannot fertilize pure *G. firmus* females (one-way incompatibility is maintained in the F1 generation), they exhibit normal fertility when mated to F1 or to G. *pennsylvanicus* females (Maroja *et al.* 2009b; L. S. Maroja and E. L. Larson, unpublished data). Given that hybrid males are fertile, and that the unidirectional barrier to gene exchange is likely due to a failure in fertilization (Larson *et al.* 2012b), mechanisms contributing to the accumulation of X-linked Dobzhansky-Mueller incompatibilities cannot explain the excess of reduced introgression X-linked loci in field crickets.

The dynamics of the cricket hybrid zone may contribute to reduced introgression of X-linked loci. Because F1 hybrids can only be produced from *G. pennsylvanicus* females (Harrison 1983), the F1 generation will exhibit a 2:1 excess of *G. pennsylvanicus* X chromosomes, whereas the autosomes will be balanced. Furthermore, because male F1 hybrids carry the *G. pennsylvanicus* X chromosome, and because these males cannot backcross to *G. firmus* females (Maroja *et al.* 2009b; L. S. Maroja and E. L. Larson, unpublished data), no X introgression from F1 males will be possible. In contrast, *G. firmus* autosomes can introgress into the *G. pennsylvanicus* background through the F1 male hybrids. Although the X chromosome could introgress into either species through F1 females, in the laboratory F1 females mate more promptly with *G. firmus* males (Maroja *et al.* 2009b). The reduced mobility of the X chromosome (introgression only possible from female F1s) might explain the steeper X-linked genomic clines observed (Larson *et al.* 2013a, 2014). Of course this hybridization dynamic does not preclude the existence of barrier genes on the X chromosome. In fact, if such genes are present, then the pattern of reduced introgression observed across the X would be enhanced. As mentioned above, the X has reduced recombination when compared to autosomes, leading to a stronger hitchhiking effect. Thus, if any loci are under selection, then X introgression will be less likely and the entire X might have restricted introgression if enough loci are under selection (C. A. Muirhead, D. C. Presgraves, unpublished data). Many previous studies have identified the X as a hotspot for genetic differentiation between species, as well as a region of the genome that is resistant to introgression (Saetre *et al.* 2003; Payseur *et al.* 2004; Carneiro *et al.* 2010; Garrigan *et al.* 2012; Sankararaman *et al.* 2014).

The only other chromosome that showed an excess of reduced introgression loci was LG14. All genes in LG14 showed reduced introgression across the Connecticut hybrid zone (Larson *et al.* 2014), and only two did not have reduced introgression in the Pennsylvania hybrid zone (possibly because of lower statistical power) (Larson *et al.* 2013a). All of these loci were in close linkage (within 7 cM), suggesting that the region could contain one or more candidate barrier genes. Genomic clines for these loci were remarkably steep and all centered at ∼310 m along the hybrid zone transect (Larson *et al.* 2014). Given the dispersal abilities of field crickets (Harrison 1979), strong selection and tight linkage across the region are required to maintain the observed steep clines. Although only autosome LG14 had many loci with reduced introgression, autosome LG2 had two genes with reduced introgression observed only in the Connecticut hybrid zone (Larson *et al.* 2014). These loci did not show reduced introgression in the Pennsylvania hybrid zone (Larson *et al.* 2013a) and other loci in close linkage (0 to 1 cM away) did not show reduced introgression in either hybrid zone. It is thus less clear how to interpret these two loci; they do not seem to represent an unambiguous region of reduced introgression.

The clustered location of highly differentiated, reduced introgression loci is suggestive of islands of restricted introgression in a background of relatively free gene exchange (“genomic islands of speciation”) (Turner *et al.* 2005). According to this interpretation, the reduced introgression loci, likely involved in reproductive barriers to gene exchange, would be unable to cross the species boundary, whereas the rest of the genome would be free to introgress, resulting in islands of divergence (Turner *et al.* 2005; Harr 2006; Nosil *et al.* 2009). The two cricket species are thought to have diverged in allopatry during the late Pleistocene, with episodic periods of secondary contact. Allopatric divergence is not expected to produce a pattern of discrete divergence islands within the genome. However, upon secondary contact, ongoing gene flow between recently diverged lineages may lead to such a pattern; the dynamics may be akin to that seen in divergence with gene flow (Via and West 2008; Feder and Nosil 2010). In such a scenario of differential introgression, only genome regions that contribute to reproductive isolation or local adaptation remain differentiated. However, similar patterns can be produced in the absence of gene flow, where a pattern of high relative differentiation (high Fst or fixed differences) could be caused by recent selective sweeps, with genomes regions that have not diverged representing the persistence of shared ancestral polymorphisms (Nachman and Payseur 2012; Cruickshank and Hahn 2014). If introgression is what is homogenizing the genomic background, then both absolute (*e.g.*, *Dxy*) and relative (*e.g.*, *Fst*) divergence in the islands should be high (Cruickshank and Hahn 2014). Our highly differentiated RNAseq loci were selected on the basis of fixed or nearly fixed differences (high *D*) between species, a relative measure of divergence. In the absence of absolute estimates of divergence (not reliably obtained from our data), regions of high differentiation could result from the reduction in genetic diversity that follows a selective event (which would increase the relative but not the absolute measure of divergence).

Both processes may be operating to produce the observed patterns. Not all loci that exhibit high *D* also exhibit restricted introgression. It is surely possible that there has not been sufficient time since the most recent secondary contact for introgression to homogenize regions across the entire species range. Furthermore, given that prezygotic isolation between these two species is strong (Harrison 1983, 1985; Maroja *et al.* 2009b; Larson *et al.* 2013a,b, 2014; Maroja *et al.* 2014), that gene flow is restricted on a fine scale (Ross and Harrison 2002; Larson *et al.* 2014), and that linkage disequilibrium is high even in the center of the hybrid zone (Harrison and Bogdanowicz 1997), it is also possible that all markers have restricted introgression to some extent. However, most individuals in the hybrid zone are multi-generation backcrosses and the extent of introgression does vary dramatically among markers (Larson *et al.* 2013a, 2014). This suggests that genome regions on LG14 and the X chromosome may indeed be contributing to reproductive isolation.

### Conclusions

Here we demonstrate that highly differentiated RNAseq loci are clustered across the genome and that reduced introgression loci are concentrated on the sex chromosome and in one small region of a single autosome (LG14). Prezygotic barriers to gene exchange, possibly combined with postmating prezygotic barriers are important in the cricket hybrid zone and in many recently diverged species. Although currently we have no evidence for the accumulation of ecological, behavioral, or fertilization prezygotic barrier genes on the X chromosome (Qvarnstrom and Bailey 2009), faster-X evolution could make these barriers more likely to be X-linked, perhaps explaining our observation of an accumulation of both high-differentiation loci and low-introgression loci on the X chromosome.

## 

## Supplementary Material

Supporting Information
