# Supplementary material for: Genes with Restricted Introgression in a Field Cricket (Gryllus firmus/Gryllus pennsylvanicus) Hybrid Zone Are Concentrated on the X Chromosome and a Single Autosome
Source: G3 (Bethesda). 2015 Aug 26;5(11):2219–27. doi: 10.1534/g3.115.021246 (PMC4632042; doi:10.1534/g3.115.021246)
Supplement: Supporting Information [file supp_5_11_2219__index.html]

Genes with Restricted Introgression in a Field Cricket (Gryllus firmus/Gryllus pennsylvanicus) Hybrid Zone Are Concentrated on the X Chromosome and a Single Autosome — Supporting Information 

# Genes with Restricted Introgression in a Field Cricket (*Gryllus firmus/Gryllus pennsylvanicus*) Hybrid Zone Are Concentrated on the X Chromosome and a Single Autosome

## Supporting Information for Maroja *et al.*, 2015

**Files in this Data Supplement:**

- Supporting Information - Tables S1-S3 (PDF, 140 KB)
- Table S2 - Information about new microsatellite loci developed for this project. (PDF, 71 KB)
- Table S1 - Genotype data used in JoinMap 4.0. (.xlsx, 180 KB)
- Table S3 - Information on previously characterized markers and their map position. (.xlsx, 16 KB)
