# Supplementary material for: Genes with Restricted Introgression in a Field Cricket (Gryllus firmus/Gryllus pennsylvanicus) Hybrid Zone Are Concentrated on the X Chromosome and a Single Autosome
Source: G3 (Bethesda). 2015 Aug 26;5(11):2219–27. doi: 10.1534/g3.115.021246 (PMC4632042; doi:10.1534/g3.115.021246)
Supplement: Supporting Information [file supp_g3.115.021246_TableS2.pdf]

Table S2 Information about new microsatellite loci developed for this project. Locus name, Genbank accession number, repeat motif, repeat number, product length (range) and primer sequence for forward and reverse primers.

Locus Name	accession number	repeat motif	repeat number	product length (bp)	Primer sequence (5'-3')
G4	KM203912	TG	15	147-205	F: GGCCATTGAATATTCTATAACCAG<br>R: AACAGCGTGAAGACTGTCTTATTT
G25	KM203913	GAT	10	248-272	F: TGCATCCCACCGGAATGAAAATC<br>R: CCGATAGCCGCGGCAGTAGTG
G11	KM203914	CA	13	367-405	F: CGGTATTGCCCAACTGCAGATTCC<br>R: ATGGGTAGTAGTGTAGTGCATGTTAAAGTAA
G7	KM203915	TTC	13	122-162	F: TTTTTCAACAACACGCCGTCAGA<br>R: TCACATCTTTATGAAAACGGGGAAATAT
G21	KM203916	CAA	14	236-245	F: TGCCGGTAGCTATCAAACACAGG<br>R: TCAAACACGAGATACATCCAGAAAGT
